# African Environmental Ethics: Keys to Sustainable Development Through Agroecological Villages

**DOI:** 10.1007/s10806-021-09853-4

**Published:** 2021-06-05

**Authors:** Charles Verharen, Flordeliz Bugarin, John Tharakan, Enrico Wensing, Bekele Gutema, Joseph Fortunak, George Middendorf

**Affiliations:** 1 Department of Philosophy, Howard University, Washington, D.C USA; 2 Department of African Studies, Howard University, Washington, D.C USA; 3 Department of Chemical Engineering, Howard University, Washington, D.C USA; 4 Center for Global Health, Old Dominion University, Norfolk, VA USA; 5 Department of Philosophy, Addis Ababa University, Addis Ababa, Ethiopia; 6 Departments of Chemistry and Pharmacology, Howard University, Washington, D.C USA; 7 Department of Biology, Howard University, Washington, D.C USA

**Keywords:** African environmental ethics, Advanced technologies, Songhaï centers, Global consortium of ecovillages, Funding initiatives

## Abstract

This essay proposes African-based ethical solutions to profound human problems and a working African model to address those problems. The model promotes sustainability through advanced agroecological and information communication technologies. The essay’s first section reviews the ethical ground of that model in the work of the Senegalese scholar, Cheikh Anta Diop. The essay’s second section examines an applied African model for translating African ethical speculation into practice. Deeply immersed in European and African ethics, Godfrey Nzamujo developed the Songhaï Centers to solve the problem of rural poverty in seventeen African countries. Harnessing advanced technologies within a holistic agroecological ecosystem, Nzamujo’s villages furnish education spanning the fields of ethics, information communication technology, microbiology, international development, and mechanical, electrical, civil and biological engineering in a community-based and centered development enterprise. The essay proposes a global consortium of ecovillages based on Nzamujo’s model. The final section explores funding methods for the consortium. The conclusion contemplates a return to Africa to supplement environmental ethics that enhance life’s future on earth.

## Introduction: Environmental Ethics in a Time of Existential Crises

This essay proposes African-based ethical solutions to profound environmental problems and a working African model to address those problems. The model promotes sustainability through advanced agroecological and information communication technologies. Climate change, the sixth mass extinction, and weapons of mass destruction threaten humanity’s survival (Gardiner, 2011; Kolbert, 2014, 2021; Schell, 2000). The zoonotic Covid-19 pandemic poses a threat of another order.

To help avert these catastrophes, we propose an African environmental ethics linked together with the physical and social sciences to confront the existential challenges to African peoples. Grounded in an African sense of communalism first expressed in ancient Egypt some five thousand years ago (Verharen et al., 2014), this ethics must be the product of all stakeholders in the communities that adopt it.

This essay’s presupposition is that the task of ethics is to issue prescriptions as to how we should live to promote life’s survival and flourishing. Unless an ethics addresses the well-being of every human as well as other organisms together with the environment that makes life possible, life as we know it will have no future on the planet.

African ethics challenges the assumption that only scientists or philosophers are competent to make ethical pronouncements. Scientists like the physicist, Stephen Hawking (2010), the biologist E.O. Wilson (2012, 2014) and the psychologist Stephen Pinker (2011, 2013, 2018) have been optimistic about science’s capacity to yield viable answers to the question of how we should live. European and North American philosophers insist that three European-derived ethical systems—utilitarianism, virtue and duty ethics—deliver viable answers to the question. The collective United Nations Declarations of Universal Human Rights propose a direction for humanity’s future that does not elaborate the means to achieve that future.

Do these ethical systems have the power to promote life’s future? After five thousand years of speculation on how we should live, perhaps one billion humans are at risk of food insecurity with their children suffering from malnutrition. As many as three billion do not have access to toilets. As many as five thousand children die daily from drinking contaminated water.

Twenty-seven of the poorest countries in the world are in Africa. According to the World Bank, if circumstances continue as expected, global poverty will be 90% African in 2030. Africa is particularly hit hard, since many of its most vulnerable people face abject, multidimensional, and chronic poverty. An estimated 40% of Africans live below 1.90 USD a day (World Bank, 2019). Beumer and Swart (2021) document the correlation between African poverty and agriculture. Covid-19 will exacerbate poverty not only in Africa but in Asia and the Americas as well.

The essay’s methodology includes a literature review of African environmental ethics together with field observations and conversations with experts in agroecology in Benin in West Africa. The essay addresses an African response to the question, “How should we live?” Populations at risk have an incentive to imagine novel answers. The essay’s first theoretical section concentrates on the ethical system proposed by the Senegalese scholar, Cheikh Anta Diop. While his broad claims about the cultural unity of Africa have been widely criticized, the essay focuses on his consilience of philosophy and science generating an environmental ethics that has the potential to confront humanity’s current existential crises.

The essay’s second section shows how a Nigerian philosopher, Godfrey Nzamujo, has developed a model that translates Diop’s ethical theory into practice. Nzamujo’s model, the Songhaï Centers, establishes ecovillages grounded in a philosophy of sustainability made possible through advanced technologies in information communication, renewable energy and agroecology. Nzamujo’s villages are rooted in African traditions, enhanced by independence from commercial fertilizers and pesticides, and interlinked with a network of Songhaï Centers and the communities in which the centers are embedded. Starting in Porto Novo in Benin, the Songhaï Center model has spread to more than fifty sites in seventeen African countries.

The essay’s next section outlines a proposal for a global consortium based on the Songhaï model of agroecological villages and facilitated through the International Network on Appropriate Technology (INAT). We examine initiatives in Africa and Asia grounded in the model’s ethical principles. Critical to an implementation of the Songhaï model is the capital required to establish a global network of ecovillages. The final section offers fund-raising proposals directed by local communities to promote a global consortium of ecovillages.

The conclusion advocates the implementation of a continental and diasporic African environmental ethics to help ensure life’s future on earth. For the first time in *Homo sapiens’* ~ 300,000 year history, technologies such as fossil fuel and weapons of mass destruction have given humanity the power to exterminate life as we know it. Advanced technologies implemented within an agroecological ecosystem have the potential to provide every human with a life of flourishing. The coronavirus pandemic and its inevitable successors demonstrate the need for a “whole Earth” response to threats to life.

A brief literature review provides examples of research on sustainable development in agroecological villages in the essay’s primary areas. A comprehensive review is a matter for another essay. Research in education for global sustainability has been converging on the model we propose in the paper (Adenle et al., 2015; Barlett & Chase, 2013; Bennett et al., 2018; Kates & Dasgupta, 2007). Extant research addresses links between environmental ethics and sustainability (Fernandes & Guiomar, 2016). The literature on agroecological villages in Africa is extensive (Bellwood-Howard & Ripoll, 2020; Brombin, 2019; Gliessman, 2018; Miller, 2018; Mousseau, 2015; Nicholls & Altieri, 2018; Pimbert, 2017; Xue, 2014). Critical to a consortium of African ecovillages is expansion of information communication technology in Africa’s poorest regions (Asonguab et al. 2018; Oladipo & Grobler, 2020; Tchamyoua et al. 2019; Wei, 2020). The content delivered by that technology is also critical. A current volume reviews the efforts of ten universities in Eurasia and South America to link higher education to sustainable development (Sabogal et al., 2020). That volume, initiated by the Free University of Berlin and The Catholic University of Peru, does not include African universities. Other research records the efforts of African universities to link higher education and sustainable development (Lotz-Sisitka, Belliethathan, et al., 2017; Lotz-Sisitka, Shumba, et al., 2017; Matiwaza & Boodhoo, 2020).

## African Environmental Ethics: Survival and Flourishing

The essay’s environmental ethics is an “African Survival Ethics” that emerges from reflection on humanity’s collective ethical systems, starting with the ancient Egyptian ethical system and concluding with recent African responses to challenges to their communities’ survival and flourishing (Verharen, 2012). Extensive literature on African environmental ethics is now available (Behrens, 2010, 2012, 2014; Bujo, 2009; Chemhuru, 2016, 2019; Horsthemke, 2005; Kelbessa, 2005, 2010, 2015a, 2015b and Masaka, 2019).

This essay focuses on the environmental ethics of the Senegalese scholar, Cheikh Anta Diop, because of his consilience of science and ethics. Initially taking degrees in philosophy and chemistry and studying with anthropologists such as Gaston Bachelard and Marcel Griaule, Diop received his doctorate at the Sorbonne in history. His education in Paris also included Egyptology, linguistics, economics, and sociology. After teaching chemistry and physics in Paris, he began to pursue studies in nuclear physics with Marie Curie’s son-in-law (Diop C.M, 2003). 

Diop exemplifies a philosopher who pursues philosophy as synoptic vision in the tradition of Plato (*Republic,* 543b) and Aristotle (*Metaphysics,* 982a9-10,), Hegel (*The Philosophy of History* 1956/1837) and Nietzsche (*The Gay Science* 2001/1887). Although STEM disciplines currently threaten philosophy and the other humanities’ futures, the idea of gathering together knowledge in all disciplines in pursuit of wisdom as total knowledge is gaining ground (National Academies of Sciences, Engineering, and Medicine 2018). Following in the footsteps of Nietzsche, the American pragmatist Richard Rorty insists that philosophy’s immortality springs from what Nietzsche calls its “mountain top” vision (Rorty, 2016, 61; Nietzsche 2014/1886, 114–115). Frodeman and Briggle (2016) call this methodology “field philosophy” with the idea of joining the physical and social sciences together with the humanities in order to address the existential crises that threaten life’s future on earth.

Anticipating the concept of field philosophy, Diop believes that a conflation of philosophy and science has the power to overcome the “barbarism” of unchecked science and technology that promises to destroy civilization’s future. In advance of this consilience, a scientist “has almost had the status of a brute, of a technician, unable to extract the philosophical importance” of scientific research. “Classical” philosophers, ungrounded in scientific research, produce metaphysical research disconnected from existential crises. Diop calls for the merger of science and philosophy “because of the one fact that the future of humanity is at stake.” Recognizing the distinction between facts and values, he insists that environmental ethics must take its foundation in ecology: “what knowledge or ‘science of the epoch’ decrees as harmful to the whole group thus becomes progressively a moral prohibition” (1991, 375). Like nineteenth century German philosophers such as Kant, Hegel, Marx and Nietzsche, Diop envisions a future where all humans are united in a single group. German optimism joins together with an “African optimism” that “inclines us to wish that all nations would join hands to build a planetary civilization instead of sinking down to barbarism” (1991, 7). He is confident that “[h]umanity’s moral conscience progresses” toward a “new perception of humanity without ethnic coordinates” by reason of a “forced progress of the world’s ethical conscience” (1991, 375–6). Such moral progress coupled with advanced technology could lead to terraforming the planets. Anticipating recent research in genetic engineering, he speculates that humans will develop the dangerous capacity to create a “super-*Homo sapiens*” that would threaten their own survival (1991, 366).

Diop’s research has been criticized for making claims about the cultural unity of the African continent in the absence of detailed research grounding that hypothesis (Appiah, 1993; Howe, 1998; Moses, 1998; Walker, 2000). He places particular emphasis on the origins of a continental cultural unity in ancient Egypt, holding that ancient Egyptian culture through its three millennial run emerged from more southerly African cultures and influenced them as well. In particular, he believed that a critical examination of ancient Egyptian culture would constitute a “necessary condition for reconciling African civilizations with history….” He insists that in a “reconceived and renewed Africana culture, Egypt will play the same role that Greco-Latin antiquity plays in Western culture” (1991, 3). Critical examination of Diop’s sweeping hypotheses about African cultural unity grounded in ancient Egypt is beyond this essay’s scope.

What is important is Diop’s hypothesis that ancient Egyptian ethics anticipates an environmental ethics grounded in both bio- and ecocentrism. Prominent Egyptologists such as Erik Hornung (2001) and Ian Assmann (2002) second his conviction. Ancient Egyptians were the first in written record to delimit the anthropomorphism and anthropocencentrism characteristic of ancient cultures.

Anticipating the pre-Socratic Greek philosophers, the five-thousand-year-old Egyptian text, the *Book of the Dead,* states that the world emerges from natural processes rather than a human-like being with an infinite power of creation. Primordial matter called *Nun* exists in a chaotic state. The substance is like water but not identical to water. *Nun* undergoes an evolutionary transformation called *Khepera* to become the sun called *Ra.*

The ordered universe emerges out of chaotic water. The principle of organization is called *Maat,* translated as harmony and balance, truth and justice. Because the chaos surrounding the universe threatens the harmony of *Maat,* humanity’s ethical mission is to promote a universal harmony that includes humans, other life forms and the earth’s inorganic features (Faulkner, 2005).

Around two millennia later, the renegade pharaoh Akhenaten in his two *Hymns to Aten* proclaimed that light known as *Aten* rather than chaotic water was the primordial substance of the universe. Like *Nun, Aten* transforms itself into the billions of other forms that constitute the universe (Hornung, 1999, 1995). Assmann calls Akhenaten’s cosmogony a “cosmotheism” (Assmann, 1996). Because humans are literally creatures of light, all humans have equal moral standing. Akhenaten promotes both bio- and eco-centrism insofar as both life and inorganic matter are manifestations of the sacred (Hornung, 1999, 2001).

Diop paints African cultural unity with a broad brush. However, his claim that an ancient Egyptian ethics of *Maat* as harmony serves as a foundation for examination of environmental ethics across other African cultures. Abundant contemporary research on Bantu ethics discloses principles of community harmony called *Ubuntu* with other variations in numerous Bantu languages (Behrens, 2010; Gyekye, 1997; Murove, 2009). Research in traditional Oromo ethics in Ethiopia discloses a principle called *Naaga* comparable to the ancient Egyptian *Maat* and translated with comparable terms (Kelbessa, 2010; Verharen, 2008).

Close examination of ancient Egyptian and Ethiopian ethics could help researchers formulate environmental ethical principles grounded in a confluence of scientific and philosophical methodology. Both systems understood ethics as a process emerging from evolutionary principles. The contemporary biologist, Edward O. Wilson, argues that the human capacity for ethical behavior emerges from a genetic trait of *eusociality,* defined as the capacity to sacrifice self-interest for the sake of group-interest in appropriate circumstances. His controversial theory conflicts with the classical Hamiltonian hypothesis that self-sacrifice for group interest extends only to genetically defined groups (Wilson, 2012, 2014). The psychologist Stephen Pinker, like Cheikh Anta Diop, claims that ethics undergoes an evolutionary progression in which the “better angels of our nature” emerge (Pinker, 2011, 2018). Contemporary research efforts to conjoin ethics and the sciences may encourage researchers to re-examine our African ancestors’ reflections while developing environmental ethics that have the promise to guarantee life’s future on earth.

## An African Agroecological Model for Translating African Environmental Ethics into Practice^1^

Immersed in the study of European and African ethics, Professor Godfrey Nzamujo, a Nigerian Dominican priest, developed a model of rural ecovillages that translates Diop’s conception of field philosophy into an instrument for sustainable development in Africa’s rural regions. Nzamujo found his inspiration for ecovillages in a Diopian conception of field philosophy (for other sources of Nzamujo’s ethical reflections, see Chardin, 1959, 2004; Du Bois, 1973; and Fanon, 1952, 2008).

In the footsteps of Diop, Nzamujo did not restrict his own education to philosophy or history. He included mathematics, computer systems, development economics and microbiology. Finishing his studies at the University of California/Irvine, Nzamujo taught at Marymount College in Los Angeles, California before returning to Africa to attack the rural poor’s existential crises (Nzamujo, 2002).

After Nigerian governments rejected Nzamujo’s petitions for land to start the first ecovillage, the Beninoise government granted him a tract on the outskirts of Porto Novo with barren soil and useless land. Translating his advanced studies into action, Nzamujo applied microbiology and biomimicry principles to restore the land to fertility without using chemicals and pesticides. Nzamujo deploys appropriate sustainable technologies within a holistic agroecological ecosystem that spans the fields of ethics, information communication, microbiology, agroecology, international development, and mechanical, electrical, civil and biological engineering in a community-centered development enterprise.

Nzamujo named his model the Songhaï Center in honor of the Songhaï Empire that took power from the Mali Empire in the early fifteenth century. The Songhaï Center in Porto Novo led to the founding of three additional sites in Benin. As a measure of its success, fifty four “centrally managed Songhaï Centers” now embody the Songhaï model in seventeen African countries including Burkina Faso, Chad, Democratic Republic of the Congo, Equatorial Guinea, The Gambia, Ghana, Guinea, Liberia, Malawi, Nigeria, Republic of the Congo, Togo and Uganda (Songhaï Center, 2018, 4).

Three ethical principles guide the development of the Songhaï ecovillages. First, the villages must be *autochthonous,* rooted in the historical traditions of local cultures (Vodouhe & Zoundji, 2013). Indigenous knowledge forms the foundation of the village (Tharakan, 2015, 2020). Humanity’s collective knowledge in the humanities, social sciences and STEM disciplines adds the superstructure. The complementarity of the old and new must be worked out to guarantee the survival and well-being of the community members. Communities that resist relevant changes to their long-standing traditions exercise autonomy at risk of disrupting the natural rhythms of their environment.

Nzamujo’s second ethical principle is that the village must be *autonomous.* Self-governance and independence depend on extensive knowledge. Nzamujo envisions the ecovillage as a “knowledge enterprise,” facilitated by information communication technology (ICT). With local knowledge and ICT access to the global pool of information, the village builds the foundation for self-reliance in producing the necessities of a flourishing life: energy, housing, clean air and water, food, healthcare and the advanced education that makes such a life possible. Nzamujo distinguishes between *autonomy* and *independence* (Nzamujo, Personal Communication, 15/05/2020)*.* His version of autonomy stresses inter-dependence over independence. The ecovillages form a network of co-operation with one another and with neighboring communities. Communities that dismiss the principle of inter-dependence introduce technologies that deplete resources and devastate their environment.

Nzamujo’s third ethical maxim is that the village must be *authentic* in two senses: ethical and practical*.* Authenticity has three distinct aspects. First, the village must be dedicated to the well-being of its residents and those of the networked ecovillages as well as the residents of neighboring communities. Second, the foundation of the village must be “authentic technologies…that generate only positive effects/benefits to the producers, consumers and the environment at the same time” (Nzamujo, Personal Communication, 15/05/2020). Such technologies enhance the flourishing of the villages and their neighbors.

**Fig. 1:**
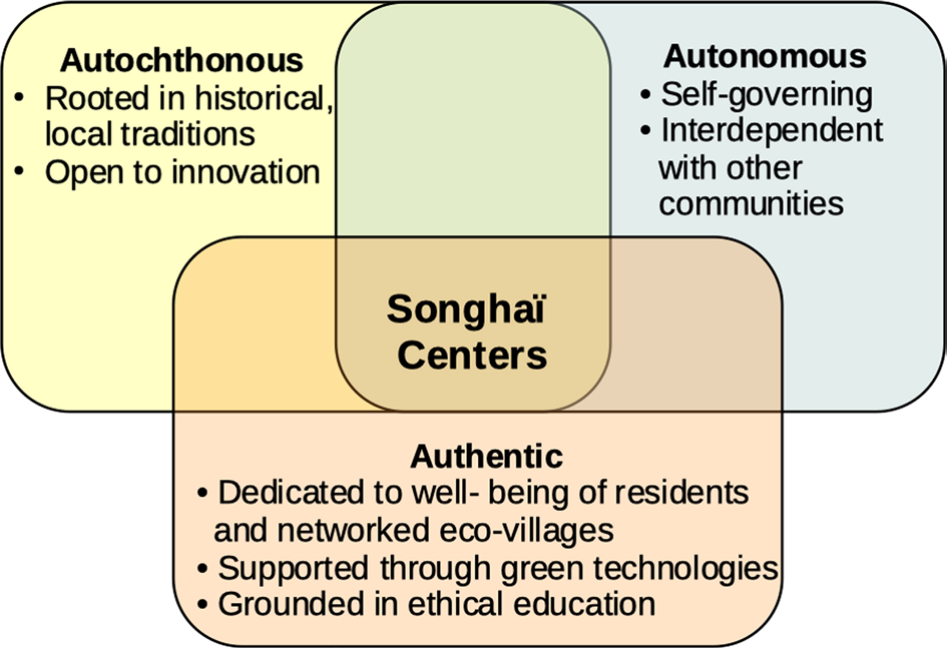
Intersection of the three ethical principles guiding the development of the Songhaï ecovillages

Third, authentic technologies are defined as those “aligned with the basic working principles of our planet” inasmuch as they “create synergy, complementarity, supplementarity, cooperation as they operate” (Nzamujo, Personal Communication, 15/05/2020). Critical to authenticity is *biomimicry* defined as the attempt to model technologies on natural processes. Authenticity includes the community’s bonding with its members, its technologies and its environment (Fig. 1).

In developing his principle of authenticity, Nzamujo, like both Teilhard de Chardin (2004/1959) and Edward O. Wilson (2012, 2014), subscribes to an ethics of moral evolution. With Chardin, Nzamujo believes that nature itself evolves from the biosphere to the noosphere—from life to life conscious of itself. A primary characteristic of self-conscious life is empathy. Like the biologist Wilson, Nzamujo holds that *Homo sapiens* has become “*Homo empathicus*” (Nzamujo, 2018, 3) In Wilson’s terms, humans like 19 other species are “*eusocial*” or genetically disposed in appropriate circumstances to sacrifice self- for group-interest.

An authentic community ensures the survival and flourishing of all its members. All in the community are guaranteed the means of survival: clean air and water, nourishing food, health care and basic education. A life of flourishing demands advanced education that enables community members to create their own choices about how to live in a community dedicated to the excellence of all its members. *Excellence* is defined by the diversity of choices that promote the future of the group’s life. In an authentic community, survival and flourishing are inter-dependent and co-evolutionary.

Authenticity includes not only community bonding principles but also community connectivity to its organic and inorganic environment through sustainable technologies. Authenticity includes three spheres: ethical, technological and environmental. The ethical addresses community bonding. The technological includes the community’s instruments for survival and flourishing. The environmental binds the community to its environment through sustainable technology.

To foster authenticity’s development Nzamujo initiated the Songhaï Leadership Academy to teach villagers and interested students how to establish their own ecovillages. The Academy offers “mathematics, statistics, agriculture (all branches), economy, environment, sociology, administrations, town planning, etc.” (Nzamujo, 2020, 31). In addition to producing scientists, engineers and technologists, the Songhaï Academy is designed to produce “spiritual leaders, mystics, wisdom writers, artists, poets, dancers, musicians, painters, philosophers” (Nzamujo, Personal Communication, 15/05/2020). Nzamujo envisions the Songhaï Centers as counterfoils to university education. He claims that “[u]niversity culture today is largely elitist” (Nzamujo, 2018, 7).

Nzamujo would like to see African universities restructuring themselves “to serve society by spurring efforts to generate knowledge, innovations, ideas and cultures commensurate with the scale, scope and complexity of the challenges that confront Africa today” (Nzamujo, 2018, 1). Nzamujo envisions the Songhaï Center as a community university that is dedicated to solving community problems. The Center curriculum is organized on the principle that “many of our technological problems have already been solved in nature in elegant, efficient and ecologically sustainable ways” (Nzamujo, 2018, 10).

The Songhaï Leadership Academy covers the humanities, social science, and STEM disciplines. Its courses are described in the *Songhaï Leadership Academy Bulletin* (Nzamujo, 2018).The program entails mastery of the techniques of organic agricultural production, including organic fertilizers, soil biology, drip irrigation, mulching, rotation, grafting, pest, parasite and predator control, agricultural machinery, animal, avian and fish husbandry, food production, storage, distribution and export from the ecovillages. Additional courses cover major aspects of ICT.

Nzamujo’s principle of authenticity enables the village to begin to be carbon–neutral through appropriate technologies such as photovoltaics, wind turbines, and the use of organic waste to produce biogas and organic fertilizer. The village offers instruction on constructing and maintaining housing for residents, students, and visitors. It also covers basic health care instruction. Specialized courses address manufacture and servicing of agricultural equipment. The Solidarity Fund for Development of French Cooperation helped the Songhaï Center in Porto Novo set up “a foundry to manufacture spare parts for agricultural machinery and food processing equipment that suit the agronomic conditions of the environment” (Nzamujo, 2018, 3).

Extensive capital investment in ICT is key to the founding of the Songhaï village. Funding from USAID helped the Songhaï Center in Porto Novo inaugurate “a network of community teleservice operations starting in 1999” and radio technology “to give the population in general and farmers in particular access to new information technologies” (Vodouhe & Zoundji, 2013, 2).

Assessment of the Songhaï Centers finds that they employ “local resources, the combination of traditional and modern agricultural practices, technology adaptation and diversification of activities” (Vodouhe & Zoundji, 2013, 3). Through its agroecological technologies, “Songhaï integrates ‘zero waste’ and ‘total productivity’ concepts” (Vodouhe & Zoundji, 2013, 3). In recognition of its successes, Benin and the United Nations contributed funding to the centers. Songhaï is acclaimed as a regional center of excellence by the Economic Community of West African States (UNDP, 2008).

**Fig. 2:**
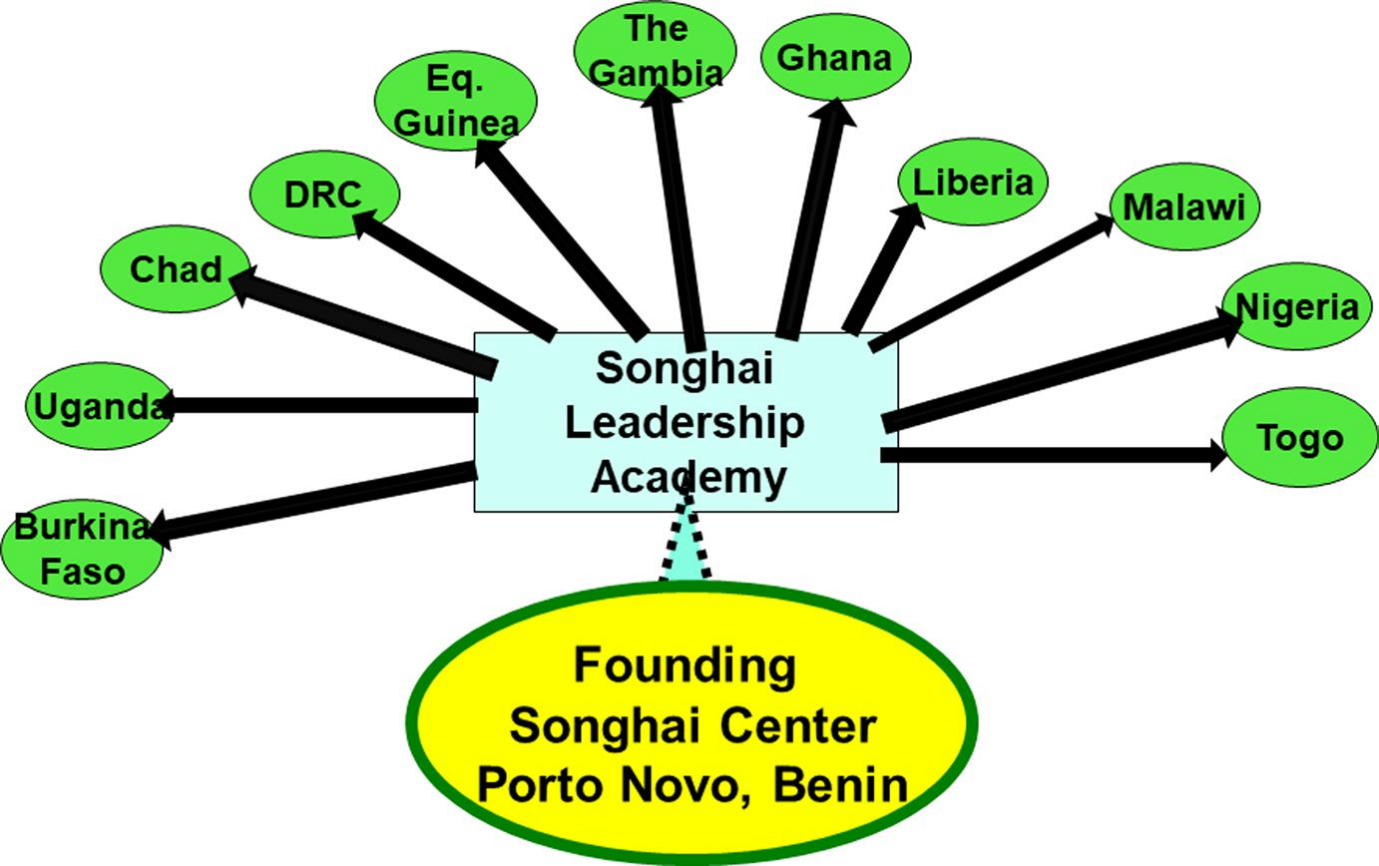
Dispersion of the Songhai model across 17 countries in Africa,including Burkina Faso, Uganda, Chad, DRC, Equatorial Guinea, The Gambia, Ghana, Liberia, Malawi, Nigeria and Togo, with over 50 ecovillage sites established

To summarize, Nzamujo’s philosophy aims to harmonize autochthony, autonomy and authenticity. While communities may ground themselves in any of these three principles, Nzamujo insists that dismissal or exaggeration of the importance of any single principle challenges community survival and flourishing. The pragmatic test of the Songhaï philosophy is its capacity for replication from the inaugural center in Porto Novo, Benin to fifty four centers in seventeen African countries. The next section explores other examples of ecovillages in Africa and India to assess whether the Songhaï model might be globalized. May a consortium of ecovillages create a global green glide path to a sustainable future for life on the planet? (Fig. 2).

## Toward a Global Consortium of Agroecological Villages Grounded in African Environmental Ethics

We propose a global consortium of groups working toward sustainable development through what Nzamujo calls authentic technologies. Many other groups throughout Africa and the rest of the Global South engage ethics to address development based on unsustainable technologies. Ecovillages are a growing global force (Brombin, 2019; Miller, 2018; Xue, 2014). The International Network on Appropriate Technology (www.appropriatetech.net) may serve as a base for establishing the global consortium. INAT members have already laid the foundation for the global consortium through their research efforts and their practical engagement with African and Indian communities. Through networking in INAT’s International Conferences on Appropriate Technology (ICAT), participants like Gada Kadoda, an independent researcher in Khartoum, have worked with Bunker Roy, the founder of the Barefoot College in India. Together, they have worked to solarize villages in Sudan. Howard University professor Joseph Fortunak will work with Godfrey Nzamujo to inaugurate production and distribution of pharmaceuticals in the Songhaï Centers. Fortunak will use the model he and his African and United States colleagues established at the St. Joseph College of Pharmacy in Tanzania (Verharen et al., 2013a).

INAT members are examining ecovillage models throughout India to determine whether their ethics would incline them to become members in a global consortium. The idea of an ecovillage has roots in the Indian independence movement with Gandhi’s call to use local resources, and develop indigenous technologies (Singh et al., 2019).

The history of the ecovillage community in India dates back to the establishment of self-sufficient Ashrams where residents grow their own food, engage in small and medium enterprises to produce goods and services, use renewable energy and aspire to zero-waste circular economies and ecosystems. An ecovillage community, the Muni Seva Ashram in Vadodaral Gujerat (www.greenashram.org), is dedicated to health care. The village includes a hospital, a cancer research and treatment center, and schools devoted to nursing, pre-K-12 education, vocational skills, and girls and women of all backgrounds. The Ashram includes a guest house for patients’ families and other guests, as well as residential facilities for the students in the various educational units.

Grounded in the principle of sustainability, the Ashram produces renewable energy through biogas, biomass gasifiers and plasma arc recycling. Both the world’s first solar crematorium and the air-conditioning systems are solar powered. While currently dependent for some power from the Vadodaral Gujerat grid, the Ashram is installing a 1.2 MW biogas plant that will render the ecovillage grid-independent and 100% renewable. Organic farming and animal husbandry provide dairy products and manure to power the biogas plants.

In Africa in The Gambia, two models embody the philosophy of the Songhaï model. The first focuses on the beekeeping industry. *BEECause Gambia*, founded in 2009 through a consortium of bee farmers, strives to use beekeeping as a method to improve the lives of the rural poor (Rahman Sallah, 2015; Africa BEECause, 2020). BeeCause Gambia regularly trains both new and old bee farmers to support sustainable bee farming. They address poverty in the region by creating new jobs while also protecting the environment through eco-friendly farming techniques. Available courses in the program include building hives, forest protection, production of bee products, and environmental conservation.

A second strategy designed to help Gambians address food insecurity revolves around the threat of global warming. To combat climate change with an early warning system, the Gambian government, United Nations Environment Programme, and other partners established fourteen pilot villages in 2014 (UN Environment Programme, 2020). Each was supplied with cell phones, radios, and loudspeakers to facilitate a mobile network. Forecasting equipment was distributed to stations to share climate information more widely. Trained volunteers transferred knowledge to community members in meetings, home visits, and “theatre” (e.g., through drama groups). With shared lessons regarding the impacts of climate change, communities have developed alternative ways of adapting. In addition to improving and developing appropriate technologies for information communication, projects have focused on community gardens that include supplemental crops to grains that rely solely on rain. They have also tried solar-powered irrigation techniques.

## African Stakeholders Directing Global North Initiatives to Capitalize Agroecological Villages

Economic resources are needed to fund agroecological start-up costs for a global consortium based on African ethics. While Nzamujo’s villages are self-sustaining once they are operational, their start-up costs are extensive, particularly with respect to information communication technology (ICT), biological laboratories, energy technologies, manufacturing tools, reservoir construction, as well as agricultural technologies such as drip irrigation, field cover and the like. Personnel training adds additional capital investment. Historical top-down funding initiatives often arise from Global North investors who direct capital flow and development technologies into unsustainable intervention. Stakeholders at the grassroots level must have a major voice in the management of funds. We propose bottom-up funding initiatives controlled by African stakeholders with emphasis on local community member engagement. Since the consortium focuses on ethical education from primary through post-secondary education, it must consider whether sufficient capital can be produced to ensure universal broadband connectivity in the Global South. The education model in Nzamujo’s Songhaï Centers is based on capital-intensive ICT.

A problem for a global ecovillage consortium is access to basic texts in all disciplines. ICT solves the problem of the immense cost of physical texts due to their production costs and publishers’ profits. A second problem is access to published research in all fields (Miguel et al. 2011; Packer, 2009, 2014). The global consortium’s task would be to encourage open source publication of basic introductory texts and a universal research library. Perhaps the most significant problem is raising the capital necessary to provide the advanced technologies that are indispensable to global ecovillages.

To promote international development that underscores the needs, voices, and perspectives of the poor, particularly in the Least Developed Countries (LDCs) in Africa, we must use the methods established by social scientists who have worked with local and indigenous communities and communities of color since the early 1900s and who regularly navigate between appropriate technologies and the most vulnerable populations in the world. Derived from applied anthropology and other social science fields (Bernard, 2013; Kedia & van Willigen, 2005; van Willigen, 2002), particularly those who work in international development (Sumner & Tribe, 2008), our proposed methods consist of the following:

First, we recommend establishing small projects that emerge from the ground up. Designed on little to no seed money, they set test conditions that allow us to determine probability of success. Faculty at universities can fund these initiatives through early concept grants, exploratory research grants, or rapid response research grants.

Second, we analyze and test the effectiveness of initial projects through a variety of methods born out of the assessment techniques used by many in international development (e.g., rapid rural and urban assessments, participatory assessments, surveys, and interviews). We collect qualitative and quantitative data that provide meaningful insights about each project. We need to use techniques that measure non-linear, multi-dimensional success that may occur in incremental steps over long periods of time.

Third, we use rigorous analysis and data to demonstrate that our innovative solutions are making a difference that outshines other initiatives. Statistical projections will be used to show that benefits from our efforts will double exponentially as we continue to establish more centers, grow networks, and launch additional projects.

Fourth, we facilitate a convergence of stakeholders and bring aboard the right actors. We ensure that our agenda and projects fit into the goals of local governments as well as different stakeholders within a community.

Fifth, we must recognize that every donor and funding organization has a unique culture. Each has mission statements, goals, and guidelines that determine the allocation of funds. Each has their own experts that abide by the social norms of their organization or discipline. Knowing the donor cultures and creating solid relationships with them will enable us to develop fundable projects that nevertheless fit into the ethical and community-driven framework.

Sixth, we must demonstrate that investment into the Songhaȉ centers serves a global benefit. We must collect data that illustrate how local challenges and hardships impact a universal collective.

**Fig. 3:**
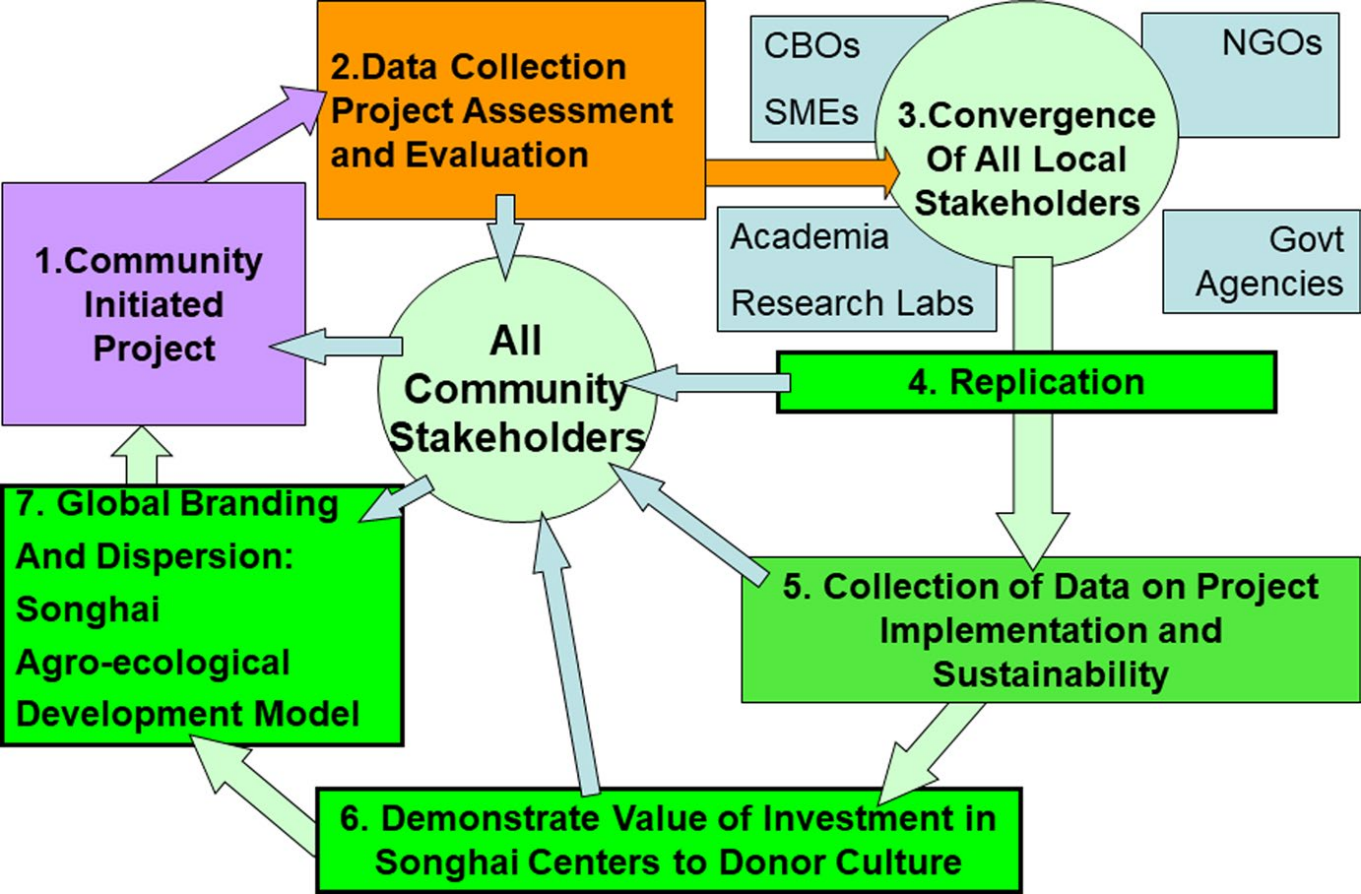
Global branding and dispersion of the Songhai Agroecological community development model using a fully integrated ethical approach to project initiation, development implementation, monitoring, evaluation and sustainable scaling

Lastly, we can create a brand, market our initiatives, and empower multiple communities to share their successes through growing networks of support. A brand can help supporters remember the Songhaȉ centers and projects and raise awareness about their significance (Fig. 3).

## Conclusion: African Environmental Ethics Linked to Agroecology for Global Survival and Flourishing

The essay’s environmental ethics is an “African Survival Ethics” that emerges from reflection on humanity’s collective ethical systems, starting with the ancient Egyptian ethical system and concluding with recent African continental and diasporic responses to challenges to their communities’ survival and flourishing (Verharen, 2012).

African ethics complements Asian and European ethical systems that have accompanied their cultures’ survival and flourishing for thousands of years. The ethical prescriptions proposed by cultures over the past five thousand years are diverse. Empirical confirmation of universal value claims is impossible. Nevertheless, nominal profession of faith in diverse ethical systems has accompanied the survival and flourishing of cultures for 3300 years for the ancient Egyptians, 3500 years for the Hindus, 2500 years for the Greeks, and 2000 years for Christians. Nineteenth century European philosophies have been tested over briefer spans, yet they have exerted global influence that shows no signs of diminishing. Our hypothesis is that such powerful and long-lasting value systems must be indispensable to survival and flourishing (Verharen et al. 2013a, b).

Ancient Africans insisted that a viable ethical prescription must harmonize conflicting values (Verharen et al., 2014). They called for a value that created harmony and balance, truth and justice. The ancient Egyptians called their single ethical value that embraced all other values *Maat* (Assmann, 1996, 2002; Verharen et al. 2014)*.*

Their ethical principle of *Maat* anticipates contemporary bio- and ecocentric proposals for granting rights not simply to humans but to other living beings and the inorganic features of the earth as well (Naess, 1989). The ancient Egyptians astound us with their cultural artifacts that have endured through the centuries (Hornung, 1999, 2001). Their greatest creation, their ethical system, joined together with the history of Africana ethical reflection, can be a guide to a global consortium of agroecological villages (Verharen, 2012).

An Africana environmental ethics can serve as the impetus for a global agroecological movement that challenges catastrophic climate change through green energy and wholesale recycling of other resources. In search of an environmental ethics that complements Asian, European and American traditions, we contemplate a return to Africa.

## Footnotes

1 This section is a revised and expanded version of earlier presentations of Nzamujo’s model in articles published in *Science and Engineering Ethics* (Verharen 2020a) and *Ethics and Education* (Verharen 2020b). Those articles focused on Nzamujo’s ethics as an example of field philosophy and his efforts to deliver ethical education to impoverished Africans. This revised and expanded section focuses on agricultural technologies appropriate for sustainable development in impoverished rural Africa. This section is intended for a different group of readers than the earlier presentations. The present article is a revised and expanded version of a paper presented at the 9th International Conference on Appropriate Technology, Tshwane University of Technology, Pretoria, South Africa, November 2020 and available in the Proceedings (https://www.appropriatetech.net).
